# Physical and Oxidative Water-in-Oil Emulsion Stability by the Addition of Liposomes from Shrimp Waste Oil with Antioxidant and Anti-Inflammatory Properties

**DOI:** 10.3390/antiox11112236

**Published:** 2022-11-13

**Authors:** Carolina Pascual-Silva, Ailén Alemán, María Elvira López-Caballero, María Pilar Montero, María del Carmen Gómez-Guillén

**Affiliations:** Institute of Food Science, Technology and Nutrition (ICTAN-CSIC). C/José Antonio Nováis 10, 28040 Madrid, Spain

**Keywords:** shrimp waste, liposomes, omega-3 fatty acids, antioxidant properties, anti-inflammatory properties, W/O emulsion, rheological properties, lipid oxidation

## Abstract

Liposomes made of partially purified phospholipids (PL) from Argentine red shrimp waste oil were loaded with two antioxidant lipid co-extracts (hexane-soluble, Hx and acetone-soluble, Ac) to provide a higher content of omega-3 fatty acids. The physical properties of the liposomes were characterized by Transmission Electron Microscopy (TEM), Dynamic Light Scattering (DLS) and Differential Scanning Calorimetry (DSC). The antioxidant and anti-inflammatory activity of the lipid extracts and liposomal suspensions were evaluated in terms of Superoxide and ABTS radical scavenging capacities and TNF-α inhibition. Uni-lamellar spherical liposomes (z-average ≈ 145 nm) with strong negative ζ potential (≈ −67 mV) were obtained in all cases. The high content of neutral lipids in the Hx extract caused structural changes in the bilayer membrane and decreased entrapment efficiency regarding astaxanthin and EPA + DHA contents. The liposomes loaded with the Hx/Ac extracts showed higher antioxidant and anti-inflammatory activity compared with empty liposomes. The liposomal dispersions improved the physical and oxidative stability of water-in-oil emulsions as compared with the PL extract, inducing pronounced close packing of water droplets. The liposomes decreased hydroperoxide formation in freshly made emulsions and prevented thio-barbituric acid-reactive substances (TBARS) accumulation during chilled storage. Liposomes from shrimp waste could be valuable nanocarriers and stabilizers in functional food emulsions.

## 1. Introduction

Shrimp processing waste oil has drawn great attention in recent years as it is an exceptional source of long chain ω-3 fatty acids (EPA and DHA) and natural antioxidants, such as α-tocopherol and carotenoids [1,2]. Processed waste from Argentine red shrimp (*Pleoticus muelleri*) represents around 45% of the initial weight and its waste oil has been reported to contain higher proportions of omega-3 polyunsaturated fatty acids (PUFAs) than that of other economically important shrimp species, such as black tiger shrimp and whiteleg shrimp [3]. Argentine red shrimp waste oil has also been regarded as a valuable source for the simultaneous extraction of natural antioxidants (α-tocopherol and astaxanthin), sterols and phospholipids (PLs) [3,4]. In particular, partially purified PLs obtained from this species were used to produce highly stable uni-lamellar nanoliposomes with a strong electronegative surface charge [4]. These ω-3 PUFA-rich liposomes were comparable in terms of particle size to those prepared with soy lecithin to encapsulate shrimp oil [5,6,7], and were smaller and more electronegative than those produced from commercial marine PLs [8]. Using higher concentrations of marine PLs to encapsulate vitamin C enhanced the free radical scavenging capacity and reducing power in the corresponding liposomes, largely attributed to the intrinsic antioxidant properties of the PLs [8]. The reported high stability of marine PLs against oxidation could be ascribed to both the content of natural co-extracted antioxidants and the PLs themselves [9,10]. In addition to the technological potential to inhibit lipid oxidation in food systems, marine PLs present considerable health effects not only associated with their intrinsic antioxidant capacity but also with their anti-inflammatory and anti-thrombotic effects [11]. Furthermore, using marine PLs in the form of liposomes has been reported to increase the intestinal bioavailability of ω-3 long chain PUFAs compared with their use in bulk oil or emulsion form, as hydrolysis by pancreatic phospholipases is slower [12].

Due to their amphiphilic nature, phospholipids are excellent emulsifying agents that can be used to stabilize food emulsions by adsorption at the oil and water interface [13]. Particularly, marine PLs present a superior emulsifying capacity than other conventional natural phospholipid emulsifiers derived from egg yolk or soybean, since their content of lyso-phospholipids, which have the ability to reduce surface tension, is higher [14].

Phospholipids are typically employed as surfactants in oil-in-water emulsions; however, in the stabilization of water-in-oil (W/O) emulsions their use is more limited due to instability issues [15]. This type of emulsion, in which water droplets are dispersed in oil, can be useful to provide semi-solid textures and to reduce fat and calorie content in certain fatty foods by increasing the dispersed phase volume fraction [16]. Several natural emulsifiers, including different lecithins, colloidal particles or polysaccharide blends have been proposed for partial or total replacement of polyglycerol poly-ricinoleate (PGPR), a commonly used synthetic surfactant in W/O emulsions [16,17]. Emulsifiers and natural antioxidants are critical factors in improving both the physical and oxidative stability of W/O emulsions, as the increased water/oil interface makes emulsions more prone to pro-oxidant attack [18,19].

Negatively charged polyethylene glycol-coated liposomes have already been proposed as particle stabilizers and delivery systems in oil-in-water food “Pickering emulsions” [20]. In this type of emulsion, iron-loaded liposomes at a high unsaturated phospholipid/iron ratio have also shown slower lipid oxidation than at a lower ratio [21]. However, the available information regarding the role of liposomes in stabilizing W/O emulsions is scarce. In any case, liposomes from marine PLs could be an excellent option to encapsulate hydrophobic antioxidant extracts rich in sterols, tocopherols and carotenoids, which, together with the additional long-chain fatty acids EPA and DHA, could proceed from the same marine raw material waste [4].

The objective of the present work was to produce liposomes rich in ω-3 fatty acids and natural intrinsic antioxidants, using partially purified phospholipids from Argentine red shrimp waste oil as the encapsulating material and lipid co-extracts from the same purification process as the different payloads. Liposomes were characterized in terms of particle properties, morphology, thermal stability, encapsulation efficiency and antioxidant and anti-inflammatory properties. The partially purified phospholipids and derived liposomes were used to explore their potential to provide physical and oxidative stability to water-in-oil emulsions.

## 2. Materials and Methods

### 2.1. Materials

Frozen Argentine red shrimp (*Pleoticus muelleri*) was purchased from a local business. Cephalothoraxes were separated, freeze-dried and subsequently ground. Partially purified phospholipids and bioactive co-extracts were obtained from the resulting powder with successive extraction steps for methanol, hexane and acetone, as described in a previous work [4]. Protein removal from the acetone-insoluble extract was carried out after alkaline solubilization. The resulting fractions were named PL, partially purified phospholipids; Hx, hexane-soluble extract; and Ac, acetone-soluble extract. The detailed chemical composition (neutral lipids, free fatty acids, phospholipids, fatty acid compositions, phospholipid species, astaxanthin, α-tocopherol, sterols, cholesterol) of PL, Hx and Ac was reported in previous work [4].

Di-sodium hydrogen phosphate dihydrate, sodium hydrogen phosphate anhydrous, hexane and acetone were purchased from Labkem S.A. (Barcelona, Spain). Sodium carbonate anhydrous, sodium hydrogen carbonate and methanol were purchased from Panreac Química S.A. (Madrid, Spain). ABTS radical was purchased from Sigma (St. Louis, MO, USA). Deionized water was obtained through the Milli-Q purification system (Merck KGaA, Darmstadt, Germany).

### 2.2. Extraction of Low Purified Phospholipids

A short lipid extraction procedure was employed, omitting the hexane solubilization step mentioned above, in order to obtain a less purified phospholipid extract for comparison purposes. In brief, 100 g of frozen shrimp powder were mixed with 350 mL of methanol (99.8%) for 16 h in darkness. The mix was centrifuged at 5500× *g* for 10 min at 4 °C and another 350 mL of methanol were poured into the pellet and mixed for another 2 h and centrifuged again. Both supernatants were poured together and centrifuged at 15,000× *g* for 20 min at 4 °C. The pellet was discarded and the supernatant was rota-evaporated completely. The resulting wax-like paste was subjected to alkaline protein solubilization to remove residual proteins and then precipitated with chilled acetone, as previously described [4]. The final product of this simplified extraction protocol involving low purified phospholipids was named PL_low_.

### 2.3. Liposome Preparation and Particle Properties

Liposomes were produced as described previously [4], using the PL fraction as the phospholipid vesicle-forming source, and the Hx and Ac extracts as the payloads. Alternatively, liposomes from PL_low_ were produced for comparison purposes. The appropriate amount of Hx or Ac (5 or 10% with respect to PL weight) was weighed and dispersed in 4 mL of 0.2 mM phosphate buffer (pH 7); then, 1 g PL was subsequently added to the dispersion. For the PL_low_ liposomes, 1 g of extract was weighed and dispersed in 4 mL of 0.2 mM phosphate buffer (pH 7). All dispersions were kept in a water bath at 60 °C for 1 h under strong stirring. Another 4 mL of phosphate buffer were added and stirred again for 1 h at 60 °C. Dispersion volumes were completed by adding 12 mL of phosphate buffer. They were subsequently vortexed for 5 min and then submitted to 5 cycles of sonication for 1 min with a 1 min pause to allow cooling in an ultrasonic cell disruptor (Model Q700, Qsonica sonicators, Newton, CT, USA) at 90% amplitude (700 W power) while kept on ice. The resulting liposomal dispersions, L-Hx_5_, L-Ac_5_, L-Ac_10_ and L-PL_low_, were stored at 4 °C for 28 days to evaluate their stability over time. For occasional comparison purposes, empty liposomes (L-E) were also produced from the PL extract following the same procedure.

A Zetasizer Nano ZS (Malvern Instruments Ltd. Worcestershire, UK) was used to measure particle size (z-average), polydispersity index (PDI) and ζ potential. All measurements were taken in quintuplicate at 25 °C after a 100-fold dilution in Milli-Q water. Measurements were performed at days 0, 14, and 28 to examine particle stability during chilled storage at 5 °C.

### 2.4. Cryo-Transmission Electron Microscopy (Cryo-TEM)

Cryo-TEM images of the fresh liposomes were taken at −180 °C using a JEOL JEM-1230 transmission electron microscope operating at 100 kV with a nominal magnification of 40 K, as previously described [22].

### 2.5. Differential Scanning Calorimetry

DSC analysis of the lyophilized liposomal dispersions was performed using a TA-Q1000 differential scanning calorimeter (TA Instruments, New Castle, DE, USA) previously calibrated by running high purity iridium (melting point, 156.4 °C; melting enthalpy, 28.44 J/g). Around 10 mg of L-Hx_5_, L-Ac_5_, L-Ac_10_ liposomes were tightly encapsulated in aluminium hermetic pans using an empty pan as reference. The samples were submitted to the following temperature cycle: starting temperature (25 °C) to −50 °C at 10 °C/min, isothermal temperature for 1 min and temperature rise from −50 °C to 110 °C at 10 °C/min under dry nitrogen purge (50 mL/min). Enthalpy (ΔH, J/g) and peak temperature (°C) were calculated using Universal V4.1D TA Instruments Software (TA Instruments, New Castle, DE, USA).

### 2.6. Antioxidant Activity

The antioxidant activity was determined in the lipid extracts and in the liposomal dispersions. Lipophilic antioxidant activity was measured by the photo-chemiluminiscence (PCL) method [23], using a PHOTOCHEM ^®^ (Analytik Jena AG, Germany) system, with the antioxidant capacity of a lipid-soluble substances kit. The instructions of the manufacturer were followed for the procedure, using Trolox as the calibration reagent. Results were expressed as mg Trolox Eq. per gram of lipid extract or L of liposomal dispersion.

Hydrophilic antioxidant activity was measured by the ABTS* radical method according to Alemán et al. [24]. ABTS* radical solution was prepared by diluting ABTS (2,2′-Azino-bis(3-ethylbenzthiazoline-6-sulfonic acid)) in a solution of 2.45 mM K_2_S_2_O_8_ up to a concentration of 7 mM; the subsequent solution was left in darkness at room temperature for 16 h and then adjusted to 0.7 absorbance at 734 nm. For radical inhibition, sample volume (20 µL) was incubated with ABTS* radical solution (980 µL) at 30 °C for 10 min and subsequently measured at 734 nm. Controls consisted of deionized water (20 µL) and ABTS* radical solution (980 µL). Results were expressed as mg Trolox Eq. per g of lipid extract or L of liposomal dispersion.

### 2.7. Astaxanthin Determination

Astaxanthin (Asx) was extracted from the PL, Hx and Ac extracts, as well as from the fresh liposomal dispersions, as previously described [25], introducing some modifications. Both lipid extracts and liposomal dispersions were mixed with hexane at a ratio of 1:1 (*w*/*v* or *v*/*v*), shaking vigorously in a vortex for 1 min, followed by centrifugation at 6000× g for 30 min at 4 °C. The supernatants obtained were measured at 470 nm in a spectrophotometer (UV-1603 Shimadzu). Astaxanthin content was determined according to Equation (1):

(1) $$\mathrm{Asx}\;\left(\mathrm{mg}\right)=\frac{A*V*P}{\varepsilon }$$

where A is the absorbance, V is the dilution volume (mL), P is the molecular weight of astaxanthin (597) and ε is the molar absorption coefficient of astaxanthin (125,100).

### 2.8. EPA and DHA Determination

Fatty acid methyl esters were determined in the PL, Hx and Ac extracts and in fresh liposomal dispersions by GC-FID, as previously described [4]. EPA and DHA were identified and quantified by comparing retention times with those of standards FAME 37 SUPELCO Ref CRM47885 + PUFA N°2 Animal Source Ref 47015-U Sigma + PUFA N°3 Menhaden oil Ref 47085-U Sigma.

### 2.9. Encapsulation Efficiency

Encapsulation efficiency (%) of the fresh liposomal dispersions was calculated using data from astaxanthin and EPA + DHA quantification, according to Equations (2) and (3):

(2) $${\mathrm{EE}}_{\mathrm{Asx}}\left(\%\right)=\frac{\left({\mathrm{Total}}_{\mathrm{Asx}}-{\mathrm{Free}}_{\mathrm{Asx}}\right)}{{\mathrm{Total}}_{\mathrm{Asx}}}*100$$

(3) $${\mathrm{EE}}_{\mathrm{EPA}+\mathrm{DHA}}\left(\%\right)=\frac{\left({\mathrm{Total}}_{\mathrm{EPA}+\mathrm{DHA}}-{\mathrm{Free}}_{\mathrm{EPA}+\mathrm{DHA}}\right)}{{\mathrm{Total}}_{\left(\mathrm{EPA}+\mathrm{DHA}\right)}}*100$$

Total Asx and total EPA + DHA were calculated, respectively, as total Asx and total EPA+DHA content determined in 1 g of PL extract plus the corresponding quantities determined in both the Hx and Ac extracts at the different concentrations added in each liposomal dispersion. Free Asx and free EPA + DHA were considered, respectively, as the Asx and EPA + DHA contents determined in the supernatants of the liposomal dispersions after solubilizing with hexane (1:1, *v*/*v*) and centrifuging at 6000× *g* for 30 min at 4 °C.

### 2.10. Cellular Viability and Anti-Inflammatory Properties

#### 2.10.1. Cell Culture

THP-1 cells were obtained from the human cell bank of the Centro de Investigaciones Biológicas Margarita Salas (CSIC) (Madrid, Spain). THP-1 cells (2 × 10^5^/mL) were maintained in an RPMI 1640 medium (Gibco, Thermo Fisher Scientific, Waltham, MA, USA) supplemented with 10% (*v*/*v*) heat-inactivated fetal bovine serum (Gibco), streptomycin (100 µg/mL) and penicillin (100 U/mL) (Gibco) at 37 °C in an atmosphere containing 5% CO_2_. They were differentiated into macrophages by adding a final concentration of 25 nM phorbol 12-myristate13-acetate (PMA) (Sigma-Aldrich, St. Louis, MO, USA) after incubation for 72 h. The cells became adherent and PMA was washed with RPMI, then the cells were cultured for 24 h in this medium.

#### 2.10.2. Cell Viability

THP-1 cells (10^5^ cells/well) in 100 μL of supplemented culture medium were added into a 96-well tissue culture plate (Falcon Microtest™, Franklin Lakes, NJ, USA) and were differentiated into macrophages as described above. Then, 10 μL of selected liposomal dispersions (L-E, L-Hx_5_ or L-Ac_10_) or 10 μL of medium (as a control) were added to the wells. Plates were incubated for 18 h, at 37 °C and 5% CO_2_. The supernatant was then removed and the cells were treated with 100 μL Krebs-Henseleit buffer (Sigma-Aldrich) and 10 μL CCK-8 (Cell Counting Kit-8, Sigma-Aldrich, St. Louis, MO, USA), according to the manufacturer’s instructions. They were incubated for 2 h at 37 °C in a dark place and relative cell viability was determined spectrophotometrically at 450 nm.

#### 2.10.3. Immuno-Stimulation

To measure the immune response of the THP-1 cells, 2 × 10^5^ cells/well in 900 μL RPMI completed culture medium were added into a 24-well plate and were differentiated into macrophages by adding PMA, as described above. Then, 100 μL of each liposomal dispersion was added and the plates were incubated at 37 °C and 5% CO_2_ for 18 h. The cells were stimulated with LPS (O26:B6, 1 μg/mL) for 4 h, before or after the addition of the samples. Two different untreated controls (non-stimulated and stimulated cells with LPS) were obtained. After the treatment, the samples were centrifuged at 1200 rpm for 5 min and the supernatants were recovered and stored at −20 °C until cytokine analysis. The concentration of each cytokine IL-10 and TNF-α released into the supernatants was quantified using an ELISA kit (Diaclone ELISA Kits, Besancon Cedex, France) according to the manufacturer’s instructions.

### 2.11. Emulsion Preparation

Water-in-oil emulsions were prepared according to Tong et al. [26] with some modifications. Commercial sunflower oil (Mercadona S.A. Spain) was mixed with the liposomal dispersions at a final ratio of 60:40, oil:aqueous phase (*w*/*w*) containing 5% of liposomes (dry matter). Another emulsion with 60% commercial sunflower oil, 5% PL extract and 35% water (*w*/*w*/*w*) was prepared as a blank. All emulsions were homogenized in cold conditions in an IKA Ultra Turrax T25 D (Staufen, Germany) at 7.2 velocity for 2 min, and immediately sonicated using a Qsonica Q700 probe sonicator (CT, USA) for 3 min at a 100% power output (700 watts), alternating 30 s of sonication and 30 s of cooling off.

### 2.12. Optical Microscopy

Emulsion samples were observed with a bright field optical microscope Nikon Optiphot (Nikon, Tokyo, Japan) after 24h of chilled storage. A droplet of each sample was placed in the glass slide, smeared gently with the help of another empty slide and covered with a cover slip. Pictures were taken with an Optikam B5 camera (Optika, Italy) and processed with the Optika Vision Lite software at 10× and 40× zoom.

### 2.13. Rheological Properties

The flow behaviour and viscoelastic properties of the emulsions were measured after 24 h of chilled storage by means of a Discovery HR10 rheometer (Waters TA Instruments, Milford, MA, USA), using the cone-plate geometry (40 mm diameter, 2° cone angle, 53 μm gap). The temperature in the lower plate was set at 25 °C.

Flow ramps were performed from 0.1 s^−1^ to 100 s^−1^ (18 min) and back to 0.1 s^−1^ (18 min), with a pre-shear step at 100 s^−1^ for 20 s in each case. Possible thixotropy (time-dependence of flow) was analyzed from the hysteresis loop between the up and down curves, using the HR10 TA Instruments Trios software. Viscosity as a function of shear rate (s^−1^) was plotted for both upward and downward ramps.

Viscoelastic properties were determined by means of frequency sweeps, which were carried out over a range of 10 to 0.1 Hz with an oscillation strain of 1%, selected from the linear viscoelastic region (LVR). The elastic modulus (G′) and viscous modulus (G′′) were plotted as a function of frequency (Hz). The rheological tests were performed at least in triplicate.

### 2.14. Evaluation of Lipid Oxidation

Primary and secondary lipid oxidation of the emulsions were determined by the content of hydroperoxides and thio-barbituric acid reactive substances (TBARS), respectively, following the procedures described by Cengiz et al. [21] with some modifications. For hydroperoxide determination, a small quantity (0.3 g) of each emulsion was taken and mixed vigorously with 1.5 mL of n-hexane:1-propanol, 3:1 (*v*/*v*). Next, 200 µL of the hexane phase were taken and mixed with 2.8 mL of methanol:1-butanol, 2:1 (*v*/*v*) and 30 µL of previously prepared Fe reagent, incubated for 20 min at room temperature and measured in a UV-1603 Shimadzu spectrophotometer (Kyoto, Japan) at 510 nm, using all reagents in the absence of the emulsion as a blank. Results were expressed as µmol of hydroperoxides per g of oil, using a calibration curve prepared with cumene hydroperoxide standard. 

For TBARS measurement, a TCA-TBA solution was prepared with 1.09 M TCA and 0.03 M TBA, and a 2% weight solution of butylated hydroxytoluene (BHT) in ethanol was prepared the day before the analysis. Both TCA-TBA and BHT solutions were mixed in a 100:3 ratio (*v*/*v*) and then added to the emulsion sample in a 2:1 (*v*/*v*) ratio. The mixture was incubated at 75 °C in a water bath for 30 min using screw-capped tubes to prevent reagent evaporation, then the tubes were allowed to cool for 10 min and centrifuged at 6700× *g* for another 10 min. TBARS were measured in a UV-1603 Shimadzu spectrophotometer (Kyoto, Japan) at 532 nm. For the blank, the TCA/TBA/BHT solution was mixed with water in a 2:1 ratio (*v*/*v*). Results were expressed as nmol of TBARS per g of oil, using a calibration curve prepared with 1,1,3,3-tetraethoxypropane. All measurements were performed on the freshly prepared emulsions (day 0) and after 30 days of chilled storage. Additionally, the effect of a heat treatment at 80 °C for 20 min was also tested.

### 2.15. Statistical Analyses

Statistical tests were performed using the SPSS ^©^ computer program, version 15.0 (SPSS IBM Statistics Software, Inc., Chicago, IL, USA). A one-way analysis of variance (ANOVA) was carried out with a Duncan test analysis. Differences between pairs of means were established using the Duncan test with a significance level set at *p* ≤ 0.05.

## 3. Results and Discussion

### 3.1. Particle Properties and Morphology

Table 1 shows particle size values expressed as z-average, the polydispersity index (PDI) and ζ potential of fresh liposome preparations stored for 28 days at 4 °C.

The size and PDI of freshly made empty liposomes from PL (≈85% phospholipids, 8.7% neutral lipids, 6.5% free FA) were previously reported to be 142 nm and 0.25, respectively, the latter increasing up to 0.30 after 28 days [4]. In the present work, the z-average of the liposomes that were loaded with the different lipid extracts varied slightly (*p* ≤ 0.05) between 143 nm in L-Hx_5_ at day 0 and 150.5 nm in L-Ac_5_ at day 28 (Table 1). These results indicate that the different payloads hardly altered the particle size, despite the different compositions of Hx vs. Ac, and different concentrations in Ac_5_ vs. Ac_10_. Briefly, the Hx extract presented around a 4-fold higher content in neutral lipids (≈382 mg FA/g), α-tocopherol (6.9 mg/g) and cholesterol (108 mg/g) than Ac; in contrast, free fatty acids (130 mg FA/g) and phospholipids (157 mg FA/g) in Ac were, respectively, 1.9-fold and 2.8-fold more abundant than in Hx [4]. The PDI was significantly lower (*p* ≤ 0.05) in L-Hx_5_, and did not change during storage, unlike L-Ac_5_ and L-Ac_10_, where a small increase (*p* ≤ 0.05) was observed. All these results suggested that the Hx extract conferred more particle size uniformity and storage stability without altering the mean size of the liposomes, largely attributed to the location of the most abundant non-polar lipid components and cholesterol in the hydrophobic interior of the bilayer membrane. In contrast, Hama et al. [27] found size variations in egg yolk phosphatidylcholine liposomes depending on the quantity and nature of the encapsulated substance, with astaxanthin presenting a greater growth in diameter than α-tocopherol. The ζ potential showed similar high electronegative values in the different suspensions, decreasing very slightly (*p* > 0.05) during storage, which denoted very high colloidal stability in all cases. The strong negative surface charge was attributed to the presence of anionic phospholipids (phosphatidylserine and phosphatidylinositol) in the PL extract used as the vesicle-forming material [4]. Other authors, namely Gulzar & Benjakul [7] and Darachai et al. [6], used synthetic phospholipids to encapsulate shrimp oil or PUFAs, the resulting liposomes having a similar particle size to the present ones, but a lower net ζ potential. In a previous work, a shrimp (L. vanamei) lipid extract encapsulated using partially purified soy phosphatidylcholine rendered slightly smaller liposomes (z-average = 102 nm; PDI = 0.25) with low electronegative ζ potential (−42 mV) [28].

When using the presumptively less purified phospholipid extract (PL_low_), little variations in ζ potential were observed, but the particle size increased up to 190.5 nm at day 0 and further to 209 nm after 28 days (Table 1). Moreover, the PDI of these liposomes was noticeably higher than that of L-Hx_5_. From the compositional point of view, the latter would be the most comparable to L-PL_low_, as it was loaded with the hexane-soluble extract, which was precisely the one that was not removed during PL_low_ extraction. Therefore, the differences between the two types of liposomes obtained highlights the importance of prior phospholipid purification when used as encapsulating material.

**Figure 1:**
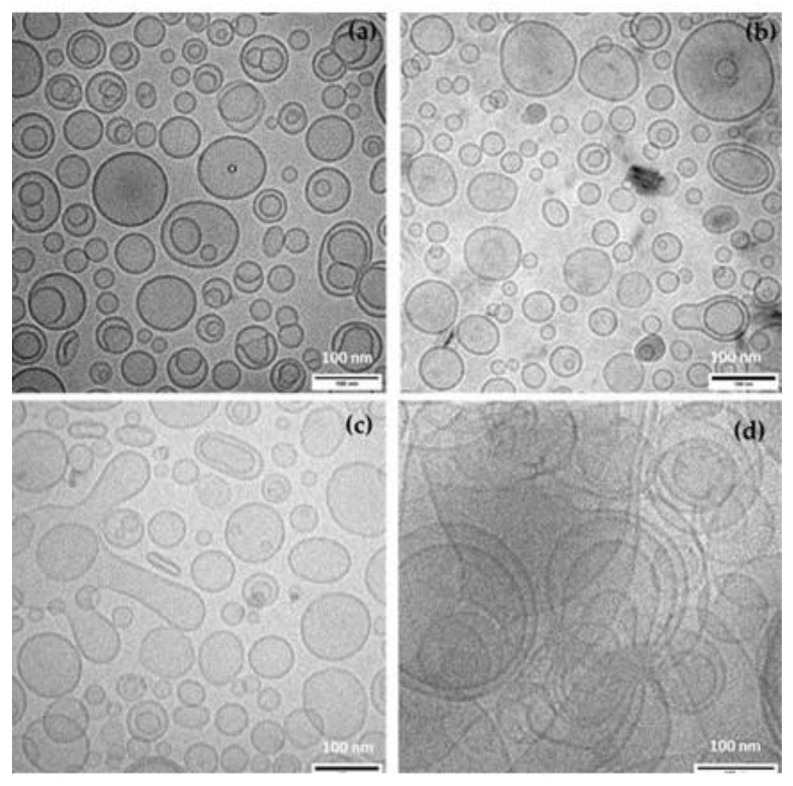
Cryo-TEM images of liposomal dispersions: (**a**) L-Hx_5_: Liposomes encapsulating 5% Hexane-Soluble Extract; (**b**) L-Ac_5_: Liposomes encapsulating 5% Acetone-Soluble Extract; (**c**) L-Ac_10_: Liposomes encapsulating 10% Acetone-Soluble Extract; (**d**) L-PL_low_: Empty liposomes made of low purified phospholipid extract.

The morphology of the studied liposomes shown in Figure 1 indicated the predominance of well-separated spherical uni-lamellar vesicles in L-Hx_5_, L-Ac_5_ and L-Ac_10_. There was a considerable lack of size uniformity, in agreement with the PDI values recorded by DLS; however, the vesicle diameters were mostly smaller than 100 nm. Round vesicles were also observed in L-PL_low_, but the image was more diffuse and larger and more heterogeneous vesicles were observed, probably indicating a greater tendency to agglomerate. Thus, in view of the generally poorer particle properties of L-PL_low_, as observed by both DLS and CryoTEM, this sample was discarded for further study.

### 3.2. Thermal Properties

**Figure 2:**
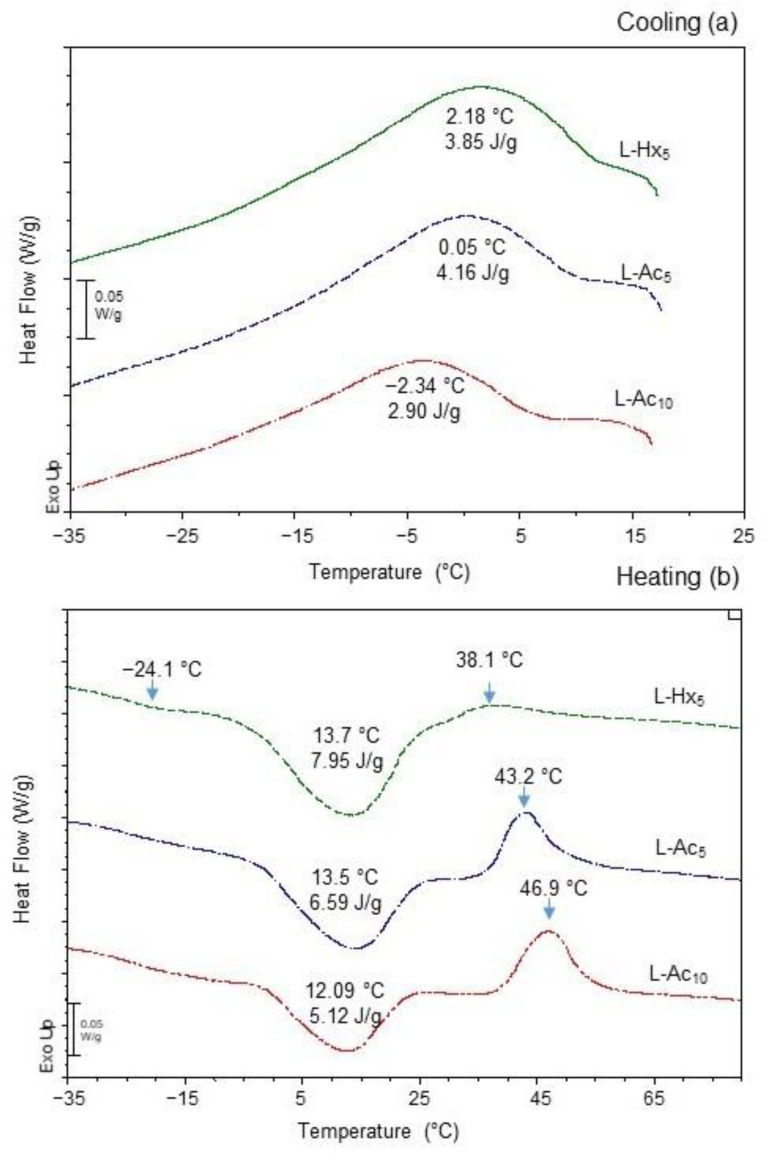
Differential scanning calorimetry (DSC) traces of lyophilized liposomal dispersions upon (**a**) cooling and (**b**) subsequent heating.

The thermal properties of L-Hx_5_, L-Ac_5_ and L-Ac_10_ during the cooling and subsequent heating ramps are shown in Figure 2a,b, respectively. In the three samples studied, the cooling behaviour was characterized by a long exothermic transition peaking at decreasing temperatures, from 2.2 °C in L-Hx_5_ to −2.3 °C in L-Ac_10_ (Figure 2a).

This thermal event was associated with the formation of a cold-induced ordered gel phase structure in the liposomal membrane. Upon subsequent heating, the main melting (gel to liquid-crystalline phase transition) temperature, as well as the associated enthalpy change (∆H), also evolved in descending order from 13.7 °C in L-Hx_5_ to 12.1 °C in L-Ac_10_, and from 7.9 J/g in L-Hx_5_ to 5.12 J/g in L-Ac_10_ (Figure 2b). These findings indicated that Hx_5_ loading provided higher liposomal thermal stability than Ac loading, which was more evident when the Ac extract was loaded at the highest concentration (L-Ac_10_). Following the main gel to liquid transition, the DSC curves of both L-Ac_5_ and L-Ac_10_ showed a pronounced exothermic event at 43.2 °C and 46.9 °C, respectively. Pascual-Silva et al. [4] reported a similar event peaking at 41.1 °C in comparable empty liposomes, which was attributed to heat-induced formation of cross-linked crystalline domains in the bilayer. This effect was considerably blurred in L-Hx_5_, which also showed a very subtle endothermic pre-transition at −24.1 °C. A sub-zero subtle quick Tg occurred in empty liposomes at a much lower temperature (−32.93 °C) [4], being attributed to probable interdigitation phenomena due to the abundance of asymmetric phospholipids rich in ω-3 polyunsaturated fatty acids [29]. The distinct DSC trace of L-Hx_5_ confirmed that the loaded extract caused noticeable structural changes in the bilayer membrane, with the slight increase in thermal stability probably being boosted by the higher amount of α-tocopherol (6.9 mg/g vs. 1.5 mg/g) and cholesterol (108 mg/g vs. 25.6 mg/g) with the Hx extract compared to Ac [4].

### 3.3. Encapsulation Efficiency (EE) and Antioxidant Activity

The different liposomal preparations were compared in terms of astaxanthin (EE_Asx_) and long-chain ω-3 fatty acid (EE_EPA + DHA_) encapsulation efficiencies (Table 2). The EE_Asx_ was 94% in L-Hx_5_ liposomes and slightly higher (*p* ≤ 0.05) in L-Ac_5_ (98.4%) and L-Ac_10_ (98.0%). Astaxanthin was also found to be highly entrapped (90%) in fresh soy phosphatidylcholine liposomes loaded with a shrimp lipid extract [5].

When EE was calculated in terms of EPA + DHA, differences related to the type of entrapped extract were more pronounced, with entrapment percentages well above 90% with the Ac extract, and considerably lower with the Hx extract (69%). The total astaxanthin content in the Ac and Hx extracts was reported to be relatively low and quite similar (1.21 and 0.98 mg/g, respectively), while the sum of EPA and DHA was insignificantly lower in Ac (130.4 and 139.5 mg/g in Ac and Hx, respectively) [4]. The lower EE_Asx_ and EE_EPA+DHA_ values in L-Hx_5_ could be due to the predominance of neutral lipids in Hx, which could saturate the hydrophobic acyl chain region of the bilayer, as suggested by DSC, and thus limit astaxanthin and EPA + DHA encapsulation. According to Hama et al. [27], the astaxanthin polyene chain tends to intercalate into the bilayer while its rings stay at the membrane surfaces. Furthermore, the greater amount of EPA and DHA in the form of free fatty acids in Ac [4] might have also favored a more efficient encapsulation. On the other hand, the two-fold increase in the Ac extract concentration in L-Ac_10_ did not substantially modify EE_Asx_ and EE_EPA+DHA_ values, therefore slightly higher amounts of both astaxanthin and especially EPA + DHA would remain non-entrapped in this sample.

The lipophilic and hydrophilic antioxidant activity of both lipid extracts and liposome dispersions were evaluated in terms of Superoxide (O_2_−) and ABTS radical scavenging capacity, respectively (Table 2). As expected, the antioxidant capacity of the studied lipid extracts in the lipophilic medium (PCL assay) was much higher than in the aqueous one (ABTS assay). In contrast, all liposomal suspensions showed a pronounced increase in ABTS values, which denotes the high capacity of liposomal encapsulation to enhance the solubility of hydrophobic compounds in aqueous media, thus improving their radical scavenging capacity and bioavailability [27,30]. The Hx extract presented PCL values that were 10 times higher than those of the Ac extract, in agreement with the higher (4-fold) content of antioxidant lipophilic compounds, such as sterols (112 mg/g) and α-tocopherol (6.9 mg/g) [4]. Interestingly, the phospholipid-rich extract (≈85% phospholipids) used as encapsulating agent also presented considerable high PCL values. Marine phospholipids, particularly those containing choline and ethanolamine, have long been known for exhibiting antioxidant activity, attributed to side-chain amino and hydroxyl functional groups [31]. In addition, PL also contained certain amounts of natural antioxidants, namely α-tocopherol (0.4 mg/g), sterols (13.3 mg/g) and astaxanthin (0.2 mg/g) [4]. Despite the great differences in PCL values between the three lipid extracts, ABTS values of liposome suspensions did not differ so much from each other, largely due to the antioxidant property provided by the PL extract in empty liposomes (L-E). In this respect, vitamin C-loaded liposomes prepared at increasing concentrations of marine phospholipids showed a progressive enhancement of their DPPH radical scavenging capacity [8]. Hx or Ac loading in the present work improved (*p* ≤ 0.05) the antioxidant capacity of the corresponding liposomes. The differences were not very pronounced, in part because the antioxidant compounds were highly entrapped, therefore loosing availability to scavenge free radicals. In the case of L-Hx_5_, the lower EE of the hexane-soluble extract could have led to a larger amount of non-polar non-entrapped compounds (tocopherol and sterols), which would explain the high PCL values. These compounds, however, were not able to increase the antioxidant property in an aqueous medium, as the lack of differences in ABTS values between L-Hx_5_ and L-E denoted. In contrast, Ac loading increased both PCL and ABTS values with respect to L-E, regardless of the concentration used. The lower PCL values with respect to L-Hx_5_ could be due to both the lower antioxidant activity of Ac in a lipophilic medium and the higher EE values. Doubling the Ac extract concentration not only did not improve the antioxidant activity of the liposomal system, but even decreased it, as shown by the significantly lower ABTS values compared to L-Ac_5_. Since EE was hardly affected by the increased Ac concentration, the lower antioxidant capacity in L-Ac_10_ could indicate slight pro-oxidant effects of certain components during the preparation of liposomes. According to Bakir et al. [32], α-tocopherol reactive radicals at high concentrations could promote lipid oxidation causing the decrease in their scavenging capacity.

### 3.4. Cellular Viability and Anti-Inflammatory Properties

Liposomes L-Hx_5_, L-Ac_10_ and empty liposomes (L-E) were selected to assess their anti-inflammatory capacity. Cell viability for all liposomal dispersions was above 95% and therefore they were considered not cytotoxic in the THP-1 cell line. An apparent viability close to 120% observed in L-Ac_10_ has been previously described in this same cell line [33] and attributed to the interference of the sample itself with the CCK-8 kit used for determination (data not shown).

**Figure 3:**
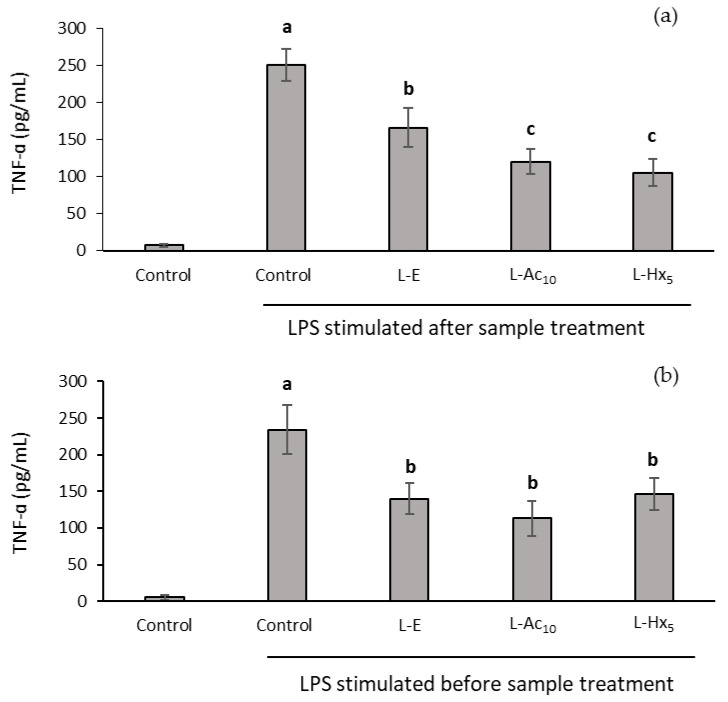
Effects of incubation with liposomes on TNF-α production in the THP-1 cells differentiated to macrophages. Cells were treated with LPS after (**a**) and before (**b**) the samples were incorporated. Data are expressed as mean ± S.D. (*n* = 6). Different letters (a,b,c) indicate significant differences (*p* ≤ 0.05) by Tukey test.

THP-1 cells were previously differentiated into macrophages to determine the anti-inflammatory capacity of the liposomes. When cells were first incubated with the liposomes and then stimulated with LPS, all samples significantly reduced the production of TNF-α factor (indicator of pro-inflammatory effect), this effect being somewhat greater in L-Ac_10_ and L-Hx_5_ (Figure 3a).

In a parallel assay, when cells were pre-stimulated with LPS and then liposomes were added, TNF-α factor production was also reduced, with a slightly greater effect than in the previous assay, demonstrating the ability of liposomes to down-regulate the inflammatory response. In this case, the activity was similar in empty liposomes (L-E) and in those in which the extracts were encapsulated (L-Ac_10_ and L-Hx_5_) (Figure 3b).

Therefore, the studied liposomes were capable of both preventing and reducing inflammation once it had started. The production of IL-10 cytokines from the liposomal dispersions (as an indicator of their anti-inflammatory capacity) was also determined before and after stimulation of the cells with LPS, but in this case the activity of the samples was practically null (data not shown).

As mentioned before, shrimp waste oil is a source of highly valuable biological molecules that may have anti-inflammatory properties. Zhang et al. [34] reported that an acetone extract of shrimp (*Pandalus borealis*) industrial waste containing mono- and polyunsaturated fatty acids (~67 and 17%, respectively) applied to human neuroblastoma SH-SY5Y cells significantly reduced the production of TNF-α and of other factors and receptors, as well as the production of reactive oxygen species. In primary cell cultures of rat alveolar macrophages, an ethanolic extract of shrimp (L. *vannamei*) waste contained carotenoids (including mainly astaxanthin) inhibited TNF-α secretion [35], an effect that was not observed when only commercial astaxanthin was added to the culture; thus, the anti-inflammatory effect was attributed to other compounds in the extract.

The anti-inflammatory properties of liposomes from marine phospholipids have been previously described. Kao et al. [36] reported that liposomes obtained from squid-skin phospholipids reduced the production of reactive oxygen species in RAW264.7 cells and the oedema produced by carrageenan administration in C57BL/6 mice. These authors concluded that liposomes act by mimicking apoptosis to resolve inflammation. Moreover, the encapsulation may improve or increase the activity of the active molecules by protecting them from the environment and by controlling their release. Gómez-Guillén et al. [25] found that a lipid extract from shrimp (L. *vannamei*) waste rich in polyunsaturated fatty acids, α-tocopherol and astaxanthin, and microencapsulated in maltodextrin by spray-drying, presented anti-inflammatory properties (measured as nitric acid production in RAW 264.7 cells), as well as antioxidant activity at concentrations ≥100 μg/mL. The free extract showed no activity, attributed by the authors to the low solubility of the samples, which may hinder its bioavailability/bio-accessibility. As previously mentioned, in the present study the extracts obtained with acetone (Ac) and hexane (Hx), as well as the partially purified phospholipids (PL) used to produce liposomes, contain astaxanthin and fatty acids of high biological value, and further in-depth studies are needed to determine the bioavailability and the fraction of these compounds that could potentially be absorbed to perform physiological functions.

### 3.5. Water-in-Oil Emulsion Stability

The ability of the different liposomal dispersions, including the empty liposomes (L-E), to improve physical and oxidative stability of W/O emulsions was evaluated, in comparison with the plain phospholipid extract (PL).

#### 3.5.1. Optical Microscopy

**Figure 4:**
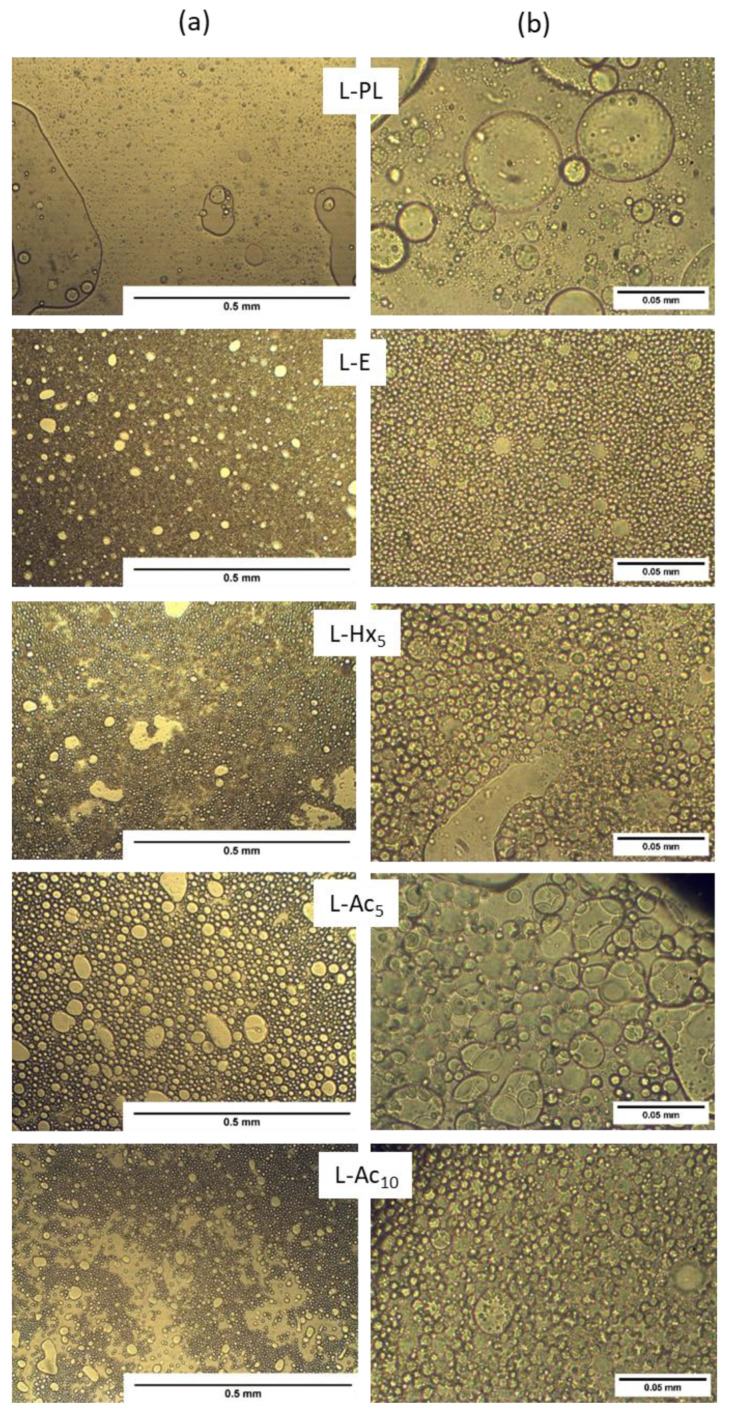
Optical microscopy of water-in-oil emulsions made with the addition of PL extract or liposomal dispersions. (**a**) 10× magnification; (**b**) 40× magnification.

The microstructure of emulsions prepared with the addition of the PL extract or the different liposomal dispersions is presented in Figure 4. The emulsion with PL showed a highly heterogeneous structure with very small droplets dispersed in a predominantly continuous oil phase. The presence of very large and irregular-shaped droplets with a thick oil/water interface was an indicator of emulsion instability, which denoted the poor emulsifying capacity of the PL extract caused by weak interface-active properties. This would probably prevent both the formation of stable water droplets and droplet coalescence during the homogenization [37]. This preparation showed an evident phase separation within the first 24 h after the production. A possible explanation could be that phospholipid concentration was not enough to produce an effective coverage at the W/O interfaces, hindering the formation of a stable well-organized emulsion [15].

In contrast, the addition of liposomes, at a similar phospholipid concentration, generally led to a pronounced increase in the mean size of the water droplets, which appeared much more homogeneous in size and more closely packed. This close packing of water droplets in a W/O emulsion can provide strong resistance to gravitational separation and increased viscosity [16]. In liposome-containing emulsions, the droplet size was noticeably smaller and more uniform in L-E, followed by L-Hx_5_ and L-Ac_5_. The droplet size in L-Ac10 decreased considerably with respect to L-Ac_5_, so that droplets prone to form large flocs caused the continuous phase of the emulsion to be clearly visible. Signs of flocculation that denoted droplet aggregation were also observable in the L-Hx_5_ emulsion. Small differences in the chemical composition and/or concentration of the loaded extracts (Hx and Ac) in liposomes, variable amounts of non-encapsulated compounds, as well as possible surface changes caused in the vesicle membranes, could induce changes in droplet size and flocculation by altering the interfacial tension and surface forces between the emulsion droplets [38].

#### 3.5.2. Rheological Properties

**Figure 5:**
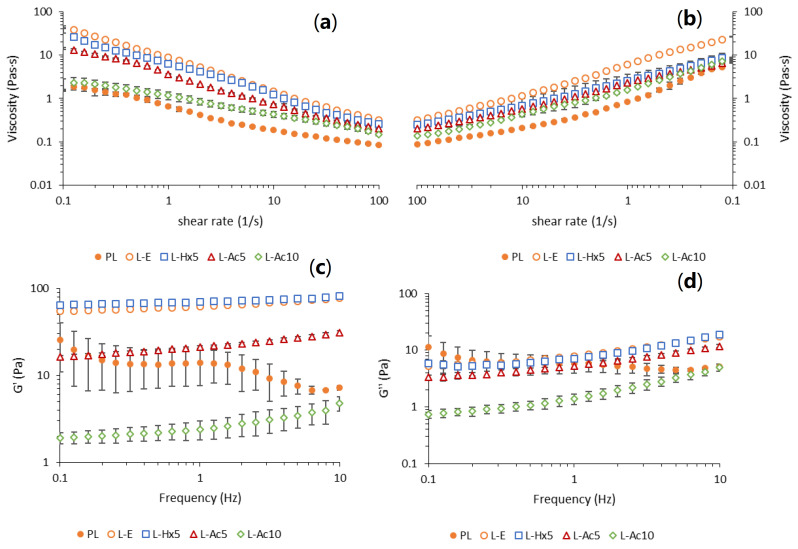
Flow behaviour and viscoelastic properties of emulsions with addition of PL extract or liposomal dispersions. Viscosity vs. shear rate in up (**a**) and down (**b**) curves; elastic modulus G′ (**c**), and viscous modulus G′′ (**d**) were plotted as a function of frequency.

The flow behavior and dynamic viscoelastic properties of the resulting fresh emulsions in which the PL extract or liposomal dispersions were incorporated are shown in Figure 5, Table 3. The up (Figure 5a) and down (Figure 5b) flow curves of all emulsion systems exhibited a typical shear-thinning response, with a decline in viscosity upon increased shear rate over the whole test range. This effect could be attributed to structural rearrangements caused by deflocculation of the emulsion droplets and the alignment along the flow direction, leading to a progressive reduction in flow resistance [39]. Noticeable differences were observed among the emulsions studied; the samples in which empty liposomes (L-E) and L-Hx_5_ were incorporated showed the highest viscosity values, particularly at low shear rates.

The viscosity (η) in up and down curves was fitted with the varying shear rate using the power law model (Equation (4)):

(4) $$\eta =\kappa \cdot {\left(\frac{d\gamma }{dt}\right)}^{n-1}$$

where κ is the consistency coefficient (viscosity value at 1 s^−1^) and *n* is the flow index (Table 3). Compared with the PL extract, the addition of liposomes generally increased the consistency of the emulsions, in agreement with the increase in the mean water droplet size that led to closer packing. This effect, which was more clearly seen in the up flow curve (κ up values), was more pronounced for emulsions in which empty liposomes were added (L-E), followed by L-Hx_5_ and L-Ac_5_. The higher emulsion structuring effect of L-E was also reflected in the lower *n* up value.

The addition of liposomes loaded with a high Ac concentration (L-Ac10) produced a pronounced destabilizing effect, as indicated by the combination of lower κ up and higher *n* up values, which were closer to that of the emulsion in which PL extract was incorporated. Comparison of up and down flow curves, lower κ down vs. κ up in L-E, L-Hx_5_ and L-Ac_5_ added emulsions, revealed the thixotropic or time-dependent shear thinning nature of these samples, which occurred more pronouncedly in L-Hx_5_. The opposite effect was observed in emulsions were PL or L-Ac_10_ were incorporated, in agreement to their lower emulsion stability. Stress vs. strain curves in up and down ramps were used to calculate the thixotropy of L-E, L-Hx_5_ and L-Ac_5_ emulsions, being 114.8 ± 10.5 Pas/s, 285.5 ± 14.8 Pas/s and 62.5 ± 11.3 Pas/s, respectively.

The increase in flow resistance in the L-E-added emulsion, compared to the rest of the emulsions containing liposomes, was largely attributed to the predominance of smaller water droplets, which favored droplet–droplet interactions by decreasing the average distance among them. In addition to droplet size, the nature of droplet interaction and droplet aggregation state could also influence the flow properties of the emulsions. Liposome particle properties (size and ζ potential) were very similar in all cases; however, the varying payload introduced slight structural changes in the liposomes, and also led to differences in non-encapsulated material, which could influence the chemical and rheological properties of the continuous phase, as well as the nature of the interfacial layers surrounding the droplet aggregates [40]. The more available phospholipid polar head groups of the L-E membrane might promote interfacial binding between the vesicles and the water/oil interface through hydrogen bonding, thus improving the emulsion stability by enhancing their adhesion to the water droplet surfaces. This interaction could be slightly impaired with L-Hx_5_ liposomes, due to the presence of non-encapsulated neutral lipids, which would also influence the rheological properties of the continuous oil phase. On the other hand, the high content of polar compounds in the Ac extract (mostly free fatty acids) could have led to a more negatively charged water droplet interphase when L-Ac_5_ liposomes were added. As a result, higher electrostatic repulsions among water droplets would have contributed to lower viscosity [41]. By increasing the Ac extract concentration when adding L-Ac_10_ liposomes, an excess of free polar compounds might have generated much smaller flocculated droplets causing higher colloidal instability. However, differences in ζ potential among the studied liposomal preparations were not significant, and the encapsulation efficiency of the Ac extract in liposomes was quite high. Thus, another possible explanation for the lower emulsion-stabilizing effects of L-Ac_5_ and especially L-Ac_10_, could be an uncontrolled damage of the liposomes during emulsion formation, which could have released part of the initially entrapped cargo [20]. The possible vesicle damage apparently did not occur in empty liposomes (L-E), and was not so evident in L-Hx_5_, due to the predominance of neutral lipids in the Hx extract and their reinforcing action of the membrane bilayer.

The dynamic viscoelastic properties of the studied emulsions, in terms of elastic modulus (G′) and viscous modulus (G′′), are shown in Figure 5c,d, respectively. All emulsions presented a predominant elastic response (G′ > G′′) in the frequency range tested. In contrast to PL, G′ and G′′ frequency dependence in liposome-containing emulsions fitted the power law model well (R^2^ ≥ 0.9) (Table 3) (Equations (5) and (6)):

G′ = G_0_′·ω*^n^*^′^ (5)

G′′ = G_0_′·ω*^n^*^′′^ (6)

where G_0_′ and G_0_′ indicated, respectively, the material resistance to elastic and viscous deformation at 1 rad/s; *n*′ and *n*′′ were the corresponding power law exponents, so that the lower the *n* value, the greater the matrix structural stability. The ratio between G′′ and G′ at 1 Hz (tanδ) is also shown in Table 3. The noticeable instability of the emulsion and the higher fluidity observed in the PL-added sample led to a much different viscoelastic behaviour, strongly influenced by the predominant continuous oil phase, which did not make power-law modelling possible in the tested frequency range.

Both emulsions L-E, and especially those with added L-Hx_5_ emulsions, showed noticeably higher G′ together with lower tanδ and *n’* values, which indicated greater predominance of the solid-like response and higher emulsion stability, attributed to the closely packed droplets producing a three dimensional structure that occupies almost the entire volume of the system [42]. The higher membrane stability of the L-Hx_5_ liposomes together with the presence of non-encapsulated neutral lipids in the continuous phase could contribute positively to reinforce the elastic character of the emulsion. Compared with samples L-E and L-Hx_5_, the mechanical spectrum and viscoelastic parameters of L-Ac_5_ denoted a weaker structure, attributed to the larger droplet size and the different water-oil interfacial tension induced by the differences in extract composition between Hx and Ac. This destabilizing effect was much more pronounced in L-Ac_10_, and the resulting emulsion also showed high fluidity. In this case, the elastic behaviour of L-Ac_10_ could come from the noticeable droplet flocculation observed in this emulsion [40].

#### 3.5.3. Oxidative Stability

**Figure 6:**
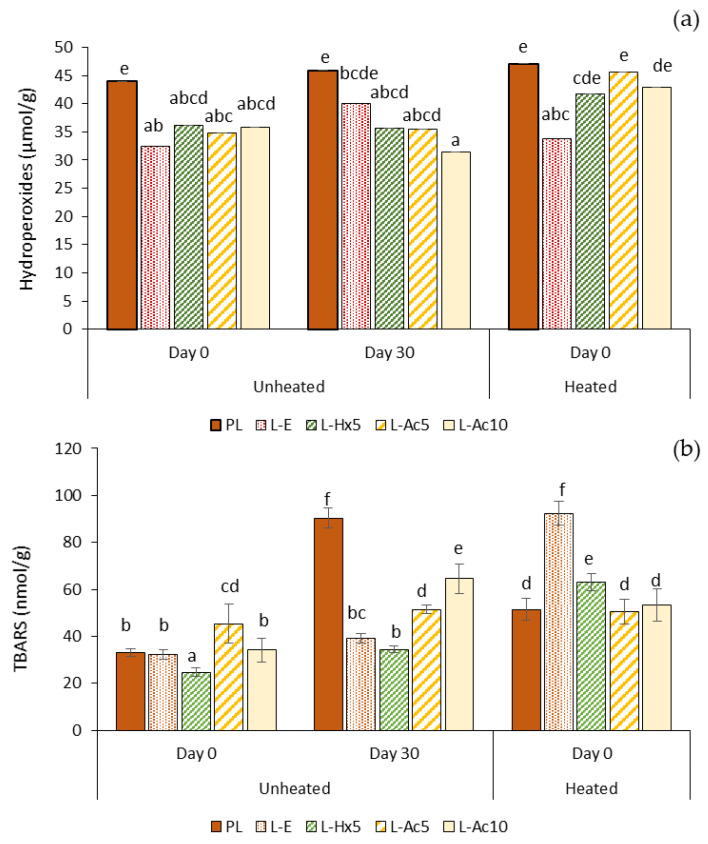
Oxidative stability in terms of (**a**) hydroperoxides formation and (**b**) TBARS accumulation in emulsions with addition of PL extract or liposomal dispersions. Different letters (a–e) indicate significant differences (*p* ≤ 0.05).

The degree of lipid oxidation was evaluated in terms of hydroperoxide (primary oxidation products) formation and TBARS (secondary oxidation products) accumulation in fresh emulsions at day 0 and after 30 days of storage at 4 °C, as well as in the emulsions heated at 80 °C (Figure 6a,b). In the freshly prepared emulsions, the hydroperoxide content was significantly higher (*p* ≤ 0.05) in the PL-added emulsion than in any of the liposome-containing emulsions, suggesting that liposomes could prevent, to a certain extent, primary oxidation during the emulsion formation. After one month of refrigerated storage no significant increase (*p* > 0.05) in hydroperoxides was observed in the different samples. The heating treatment caused hydroperoxides to increase slightly (*p* < 0.05) in emulsions L-Ac_5_ and L-Ac_10_, indicating a lower protection with these liposomes, likely associated to the joint effect of the lower physical emulsion stability and the lower antioxidant activity of Ac extract and L-Ac liposomes (PCL values) compared to the Hx counterparts.

Slight differences in TBARS content were observed in the freshly prepared emulsions; L-Hx_5_ presented the lowest (*p* ≤ 0.05) value, in agreement with the noticeably higher antioxidant capacity of the Hx extract and L-Hx_5_ liposomes. After 30 days of storage, TBARS content increased much more pronouncedly (*p* ≤ 0.05) in the emulsions PL and L-Ac_10_, which coincided with the lower physical stability of these samples. This effect could not be directly related to a lower antioxidant capacity of the PL extract or the L-Ac_10_ liposomal system, as the two performed very differently in terms of PCL or ABTS values.

Thus, it might be more plausible that the close packing of water droplets providing greater stability in emulsions L-E, L-Hx_5_ and, to some extent, also in L-Ac_5_, could have played a key role in preventing lipid oxidation during storage [18,19]. The heat treatment caused TBARS to increase in all samples, except in L-Ac_5_ where differences were not significant (*p* > 0.05). The rise in TBARS in the L-E added emulsion was especially remarkable, followed by far by that of L-Hx_5_. A possible explanation is that heating caused emulsion destabilization, and the antioxidant property of the liposomal systems in the lipophilic medium (PCL value) was not effective enough to prevent heat-induced lipid oxidation. This effect was more pronounced in the L-E-added emulsion, which had the highest initial physical stability and the lowest liposomal PCL value. Thus, the physical stability provided by ω-3-rich liposomes to W/O emulsions could prevent lipid oxidation during the emulsion formation and subsequent storage. Loading the liposomes with natural antioxidants would contribute to mitigate the pro-oxidative effect of a heat treatment.

## 4. Conclusions

Liposomes made of partially purified phospholipids from Argentine red shrimp waste can be loaded with lipid co-extracts (Hx and Ac) to produce liposomes further enriched in ω-3 fatty acids with improved antioxidant and anti-inflammatory properties. The different payloads hardly altered the particle size and surface charge of the resulting liposomes; however, the predominantly non-polar nature of Hx induced structural changes in the bilayer, leading to a lower encapsulation efficiency of EPA + DHA. In contrast to the plain phospholipid extract, the liposomal dispersions produced stable W/O emulsions with homogeneous and closely packed water droplets. Liposomes loaded with Hx increased the consistency and solid-like behaviour of the corresponding emulsions to a greater extent than with Ac. In addition to the higher membrane stability of the former, the presence of non-encapsulated neutral lipids in the continuous phase would be a key factor to reinforce the viscoelastic properties of the emulsion. The physical enhancement of W/O emulsion by the addition of liposomes with antioxidant properties provided oxidative stability during chilled storage and upon heating. These liposomes could have great potential as emulsion stabilizers in the formulation of functional foods, as for example, sauces, creams, etc.

## Figures and Tables

**Table 1:** Z-average, polydispersity index (PDI) and ζ potential of liposomes.

	Sample	0 Days	14 Days	28 Days
z-average (nm)	L-Hx_5_	143.1 ± 2.1 ^A/a^	145.7 ± 1.4 ^A/a^	144.9 ± 0.3 ^A/a^
	L-Ac_5_	145.3 ± 0.9 ^A/a^	145.9 ± 1.8 ^A/ab^	150.5 ± 2.4 ^B/b^
	L-Ac_10_	147.1 ± 2.2 ^A/b^	149.2 ± 0.5 ^A/b^	148.3 ± 1.4 ^A/ab^
	L-PL_low_	190.5 ± 1.7 ^A/c^	198.7 ± 2.3 ^B/c^	208.6 ± 2.4 ^C/c^
PDI	L-Hx_5_	0.17 ± 0.01 ^A/a^	0.18 ± 0.03 ^A/a^	0.17 ± 0.02 ^A/a^
	L-Ac_5_	0.24 ± 0.02 ^A/b^	0.25 ± 0.01 ^A/b^	0.28 ± 0.02 ^B/b^
	L-Ac_10_	0.24 ± 0.01 ^A/b^	0.27 ± 0.01 ^AB/b^	0.27 ± 0.02 ^B/b^
	L-PL_low_	0.22 ± 0.01 ^A/b^	0.21 ± 0.02 ^A/a^	0.22 ± 0.02 ^A/c^
ζ Potential (mV)	L-Hx_5_	−67.1 ± 0.9 ^A/a^	−64.1 ± 1.6 ^B/a^	−64.1 ± 1.6 ^B/a^
	L-Ac_5_	−66.9 ± 0.6 ^A/a^	−63.1 ± 1.5 ^B/a^	−56.3 ± 0.7 ^C/b^
	L-Ac_10_	−68.4 ± 0.8 ^A/ab^	−64.5 ± 1.6 ^B/a^	−63.4 ± 1.2 ^B/a^
	L-PL_low_	−69.4 ± 1.4 ^A/b^	−75.2 ± 0.9 ^B/b^	−66.8 ± 2.1 ^C/c^

L-Hx_5_: Liposomes encapsulating 5% Hexane-Soluble Extract; L-Ac_5_: Liposomes encapsulating 5% Acetone-Soluble Extract; L-Ac_10_: Liposomes encapsulating 10% Acetone-Soluble Extract; L-PL_low_: Empty liposomes made of low purified phospholipid extract. Different letters (A,B,C…) indicate significant differences (*p* ≤ 0.05) as a function of days of storage. Different letters (a,b,c…) indicate significant differences (*p* ≤ 0.05) as a function of type of sample.

**Table 2:** Encapsulation efficiency (%) of astaxanthin (EE_Asx_) and long-chain ω-3 fatty acids (EE_EPA+DHA_); and anti-oxidant capacity of lipid extracts (mg Trolox Eq/g) and fresh liposomes (mg Trolox Eq/L).

	EE_Asx_	EE_EPA+DHA_	PCL	ABTS
PL	-	-	189 ± 2.83 ^a^	3.26 ± 0.07 ^a^
Hx	-	-	596 ± 96.8 ^b^	2.53 ± 0.05 ^b^
Ac	-	-	58.2 ± 2.90 ^c^	2.24 ± 0.43 ^b^
L-E	-	-	6.68 ± 1.58 ^a^	209 ± 14.5 ^a^
L-Hx_5_	94.0 ± 0.10 ^a^	69.0 ± 0.81 ^a^	25.9 ± 1.53 ^b^	207 ± 7.66 ^a^
L-Ac_5_	98.4 ± 0.30 ^b^	95.8 ± 5.99 ^b^	12.2 ± 2.81 ^c^	245 ± 5.62 ^b^
L-Ac_10_	98.0 ± 0.13 ^b^	91.1 ± 12.6 ^b^	11.9 ± 3.22 ^c^	228 ± 1.02 ^c^

Different letters (a,b,c…) indicate significant differences (*p* ≤ 0.05). Statistical analysis for lipid extracts and liposomal dispersions has been performed separately.

**Table 3:** Flow and viscoelastic parameters for water-in-oil emulsions stabilized with partially purified phospholipids extract (PL) and liposomal dispersions.

	κ_up_ (Pa·s^−n^)	n_up_	κ_down_ (Pa·s^−n^)	n_down_	G_0_′ (Pa)	G_0_′ (Pa)	*n*′	*n*′′	tanδ
PL	0.65 ± 0.03	0.51 ± 0.02	1.04 ± 0.01	0.37 ± 0.02	-	-	-	-	0.43 ± 0.05
L-E	8.43 ± 0.24	0.26 ± 0.02	5.88 ± 0.03	0.33 ± 0.01	55.1 ± 2.37	5.24 ± 0.32	0.07 ± 0.01	0.26 ± 0.01	0.13 ± 0.01
L-Hx_5_	6.19 ± 0.40	0.28 ± 0.05	2.59 ± 0.94	0.45 ± 0.01	64.9 ± 1.92	4.90 ± 0.32	0.04 ± 0.01	0.27 ± 0.03	0.10 ± 0.01
L-Ac_5_	3.56 ± 0.11	0.34 ± 0.00	2.16 ± 0.01	0.46 ± 0.01	16.8 ± 0.66	3.34 ± 0.39	0.14 ± 0.01	0.28 ± 0.02	0.25 ± 0.03
L-Ac_10_	1.10 ± 0.22	0.59 ± 0.03	1.78 ± 0.02	0.40 ± 0.01	1.87 ± 0.32	0.76 ± 0.18	0.17 ± 0.03	0.42 ± 0.01	0.58 ± 0.02

## Data Availability

Data are contained within the article.
